# Dual-pump Kerr Micro-cavity Optical Frequency Comb with varying FSR spacing

**DOI:** 10.1038/srep28501

**Published:** 2016-06-24

**Authors:** Weiqiang Wang, Sai T. Chu, Brent E. Little, Alessia Pasquazi, Yishan Wang, Leiran Wang, Wenfu Zhang, Lei Wang, Xiaohong Hu, Guoxi Wang, Hui Hu, Yulong Su, Feitao Li, Yuanshan Liu, Wei Zhao

**Affiliations:** 1 State Key Laboratory of Transient Optics and Photonics, Xi’an Institute of Optics and Precision Mechanics (XIOPM), Chinese Academy of Sciences (CAS), Xi’an 710119, China; 2 University of Chinese Academy of Sciences, Beijing 100049, China; 3 China-UK Joint Research Center on Micro/Nano photonics, XIOPM of CAS, Xi’an 710119, China; 4 Department of Physics and Materials Science, City University of Hong Kong, Hong Kong, China; 5 Department of Physics and Astronomy, University of Sussex, Falmer, Brighton BN1 9QH, UK

## Abstract

In this paper, we demonstrate a novel dual-pump approach to generate robust optical frequency comb with varying free spectral range (FSR) spacing in a CMOS-compatible high-Q micro-ring resonator (MRR). The frequency spacing of the comb can be tuned by an integer number FSR of the MRR freely in our dual-pump scheme. The dual pumps are self-oscillated in the laser cavity loop and their wavelengths can be tuned flexibly by programming the tunable filter embedded in the cavity. By tuning the pump wavelength, broadband OFC with the bandwidth of >180 nm and the frequency-spacing varying from 6 to 46-fold FSRs is realized at a low pump power. This approach could find potential and practical applications in many areas, such as optical metrology, optical communication, and signal processing systems, for its excellent flexibility and robustness.

Optical frequency comb (OFC) characterized by discrete and equally spaced frequencies has numerous applications in the fields such as optical synthesis and high accuracy optical metrology^1^,^2^, molecular spectroscopy^3^, multichannel generators in telecommunications^4^,^5^, arbitrary optical waveform generation^6^,^7^, and optical-microwave signal processing^8^,^9^,^10^. Enormous development has been achieved concerning the OFC generation, e.g., the traditional method based on femtosecond mode-locked laser which can provide millions of lines with relative accuracies at the 10^−19^ level^11^,^12^, and can offer an octave-spanning bandwidth spectrum. However, the frequency spacing is severely limited by its low repetition rate that is depended on the laser cavity length (typical <10 GHz). So, a Fabry-Perot filter-cavity is needed to extend OFC’s frequency spacing to meet the application in arbitrary optical waveform generation^13^ and astronomical spectroscopy^14^. To get large frequency spacing OFC, modulator approach and micro-cavities scheme are two typical candidates. For the modulator type, the bandwidth of radio frequency (RF) sources and modulator is the bottleneck issue. Another problem is that the cost would increase dramatically with bandwidth growth. So the micro-cavity-based Kerr comb is the most attractive solution in ultra-high bandwidth OFCs generation and have developed rapidly in recent years for its compactness and low-cost advantage. By now it has been realized in various platforms, including crystalline resonators^15^, CMOS-compatible platforms such as silicon nitride (Si_3_N_4_)^5^,^16^,^17^, high-index doped silica glass^18^,^19^, as well as aluminum nitride^20^ and diamond^21^.

Most Kerr combs are pumped by resonantly coupling external continuous-wave (CW) laser into a high-Q nonlinear micro-cavity. The instability of locking between the external pump and resonances of the micro-cavity is a common issue faced by related experiments. In general, soft-thermal locking is exploited to solve this issue by slowly tuning the pump wavelength into the resonance, until locking is finally achieved. However, this method is relatively complex and with poor robustness to external disturbances such as slow temperature variations and pump power instabilities^19^. Meanwhile, the frequency spacing of these OFCs is the FSR of the MRR in most cases. In order to realize OFC with varying frequency spacing, several approaches have been proposed. One reported scheme is detuning the pump wavelength, and the resonant wavelength of an aluminum nitride MRR with 435 GHz FSR and 1-FSR, 2-FSR and 6-FSR frequency spacing OFC are generated^20^. Another scheme is using MaF_2_ whispering gallery mode (WGM) resonator by tuning the pump laser with respect to the corresponding resonator mode and OFCs are obtained with frequency spacing from 1 to 3-FSR^22^,^23^. Concerning the Si-based platform which takes advantage of cost-effective and excellent compatibility of the widespread CMOS technique, an impressive achievement shows that OFC with varying frequency spacing can be realized in dual-coupled Si_3_N_4_ micro-rings. By controlling the mode interactions of the dual rings through the micro-heater fabricated to the auxiliary ring, 1-FSR (378 GHz) to 5-FSR (1.89 THz) frequency spacing OFCs are obtained^24^. It is worth to note that only a few FSRs can be tuned using a single pump for these schemes mentioned above. In order to vary the OFC frequency-spacing freely, dual-pump scheme is an optimal approach which has been used to generate parametric frequency combs in both cavity-less and micro-cavity schemes. For example, Y. H. Li *et al*. presented cascaded four-wave mixing OFC pumped by two synchronized picosecond lasers at about 850 nm in optical microfibers with maximal frequency spacing up to 24 THz^25^. Z. Tong *et al*. had also demonstrated 200 GHz and 400 GHz spacing OFC generation in multistage high nonlinear fiber mixer driven by two pumps^26^. Aku A. *et al*. studied the two pump tunable OFC generation and its temporal evolution in normal dispersion fiber by numerical simulations^27^. For micro-cavities, theoretical analyses have been studied for dual-pump Kerr frequency combs generation in both normal and anomalous dispersion regimes^28^,^29^. And dual pump degenerate Kerr oscillator in silicon nitride MRRs have been theoretical and experimental studied^30^. These studies show that the pump threshold can be efficiently reduced by using dual-pump OFC generation scheme instead of the single-pump approach. Meanwhile, an OFC generation experiment demonstrated the thresholdless OFC generation in a whispering gallery mode resonator pumped by two frequencies^31^. Up to 10-fold FSR (135.6 GHz) spacing OFC is obtained with less than 20 nm bandwidth. The narrow bandwidth is caused by low pump power which is limited by the thermal effect due to a serious resonances shifting induced by the dual pumps. The thermal effect leads extremely difficult to precisely control the pump detuning, and turns to be a major obstacle to the broadband OFC generation through dual-pump scheme based on micro-resonators.

In this paper, a self-locked dual-pump OFC generation approach in a CMOS-compatible high-Q MRR with varying frequency spacing is demonstrated for the first time. The MRR is fabricated by high-index doped glass platform whose process is completely CMOS compatible, and low power CW pump four-wave mixing (FWM) has been demonstrated in this platform^32^. In our scheme, dual-pump is self-oscillated in the laser cavity loop rather than coupling external pumps to the MRR. Through employing the self-locked technique, the system can be stabilized via an intrinsic feedback mechanism which effectively acts against thermal or mechanical fluctuations in the ring parameters^19^. By tuning the pump wavelength through programming the tunable double passbands filter, broadband OFCs with frequency spacing varying from 6 to 46-fold FSRs are successfully achieved in the experiment. This proposed approach could find potential and practical applications in optical metrology, WDM optical communication systems, arbitrary optical waveform generation and optical signal processing for its robustness and flexibility.

## Results

### OFC experiment setup

**Figure 1:**
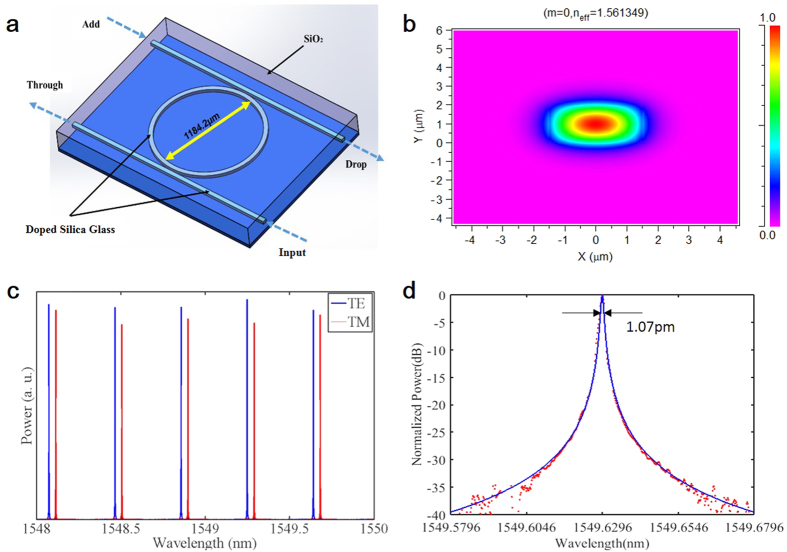
Device schematic and propagation characteristics. (**a**) Schematic of the four-port high-Q MRR. The waveguide core is a CMOS-compatible high-index doped silica glass. (**b**) Calculated mode profile for TE polarization. (**c**) Linear input-drop transmission characteristic of the ring resonator for TM and TE polarizations. (**d**) High-resolution resonance profile with a Lorentzian fit showing a 1.07 pm FWHM.

The key component of the Kerr OFC generation system, the micro-ring resonator, shown in Fig. 1, is an integrated MRR fabricated in a CMOS-compatible high index doped silica glass platform^18^,^32^ with 2 × 3 μm waveguide cross-section and 592.1 μm radius. The gaps between the bus waveguides and the resonator are 1μm. The calculated mode profile of transverse electric (TE) polarization is depicted in Fig. 1(b). The input-drop transmission spectra with a FSR of 49 GHz for both the TE and TM modes are shown in Fig. 1(c). The typical full-width at half-maximum (FWHM) is ~1.07 pm, yielding a Q-factor of about 1.45 × 10^6^ as shown in Fig. 1(d).

**Figure 2:**
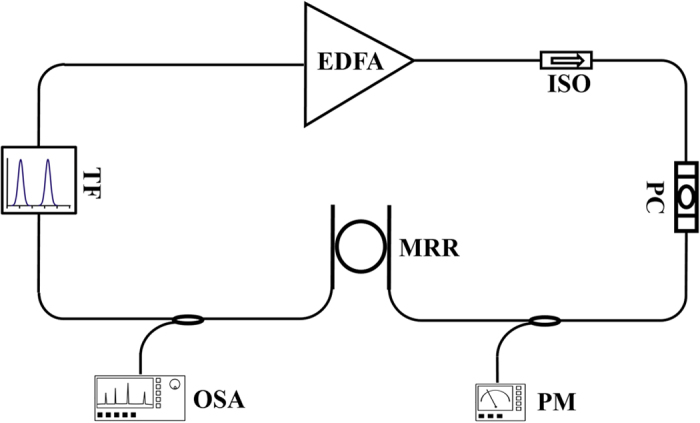
Experimental set-up for the self-locked tunable OFC generation. EDFA, erbium-doped fiber amplifier; ISO, Isolator; PC, Polarization Controller; MRR, Micro-Ring Resonator; TF, Tunable Filter; OSA, Optical Spectrum Analyzer; PM, Power Meter.

The experimental schematic is shown in Fig. 2. The MRR embeds in the main fiber loop cavity. It can not only work as a finesse filter to select oscillation frequencies together with the tunable filter in the loop, but also enhance the field interaction and contribute to new frequency generation. The main cavity contains an erbium-doped fiber amplifier (EDFA) acting as the gain medium, an isolator to ensure light unidirectional propagation in the loop cavity, a polarization controller (PC) to adjust the polarization to a single waveguide mode, and a programmable filter to select dual-pump wavelength. The programmable filter used in our experiment is a commercial filter (WaveShaper 4000 s multiport optical processor fabricated by Finisar) with bot wavelength and bandwidth tunable in C band. The optical power coupled to the bus waveguide is monitored by a power meter, and the optical spectrum is test by an OSA at the DROP port of the MRR through an 8:2 splitter. The total main cavity length is more than 40 meters, yielding a FSR of about 4.7 MHz.

### FWM in the high-Q MRR

**Figure 3:**
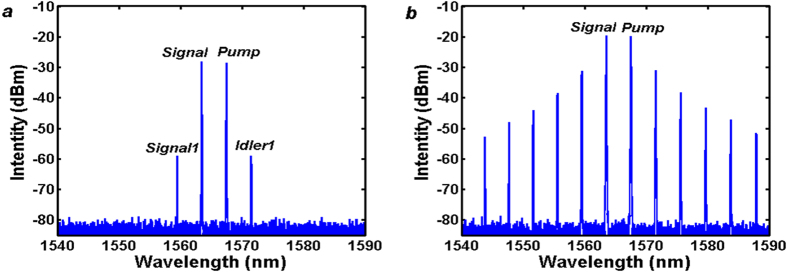
Experimental results for FWM in MRR with 490 GHz frequency spacing (10FSRs). (**a**) −1 dBm, (**b**) 10 dBm.

By programming the tunable filter, two passbands located at 1563.5 nm and 1567.5 nm are selected both with 50 GHz bandwidth. Two oscillation wavelengths are determined together with the filter characteristic of the MRR with 10-fold MRR FSR frequency spacing. Since the mode competition is effectively suppressed by the nonlinear interaction in the MRR, stable dual wavelengths oscillation can be achieved in our experiments. The lasing power of these two wavelengths is equalized by adjusting the related attention using the tunable filter. When the pump power inside the input bus waveguide reaches −1 dBm (the laser just starts lasing stably), two new frequencies located at 1559.5 nm and 1571.5 nm (denoted as signal1 and idler1 respectively) are generated via FWM in the MRR, as shown in Fig. 3(a). That shows a very low threshold FWM in the high-index platform MRR which is consistent with the report in the paper by Ferrera, M. *et al*.^32^. Then the comb-like spectrum formed by the cascaded FWM can be observed by increasing the pump power. Figure 3(b) shows the comb-like spectrum when the pump power reaches 10 dBm. At this stage, the conversion efficiency (signal1/signal) is about 8.27% and can be further improved by increasing the pump and signal power. Complex nonlinear interaction in both fiber laser cavities and the MRRs may entrain growth while raising the laser radiation power. For example, femtosecond noise-like oscillations was observed in a Yb-doped fiber laser due to the effect of non-linear polarization evolution^33^. And the spatio-temporal evolution arising from nonlinear interaction, scattering, amplifier noise and other effects in a partially mode-locked fiber laser had been experimental studied by an intensity autocorrelation function evolution mapping technique^34^. In this OFC generation experiment, we mainly focus on the FWM effect which dominants the dual-pump OFC generation.

The above experiment results shows very high efficiency FWM in the MRR. This is contributed by the high nonlinear parameter of the waveguide and enormous field enhancement in the cavity. The nonlinear parameter of the waveguide (mode field size ~4.8 μm^2^) is estimated to be about 90 times larger than standard single-mode telecommunication fibers^32^. And the theoretical model for the field enhancement factor of MRR is shown in Equation (1)^32^,^35^,


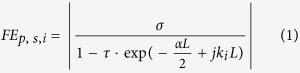


where *FEp*, *FEs* and *FEi* describe the field enhancement factor for pump, signal and idler in the MRR, respectively, L is the MRR perimeter, *α* is the linear propagation loss coefficient, 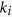 is the propagation constant in waveguide, σ and τ are the ring coupling and transmission coefficients respectively (|*σ*^2^| + |τ^2^| = 1). The overall field enhancement factor defined as (*FEp*)^4^ * (*Fes*)^2^ * (*FEi*)^2^ is calculated as ~9.14 × 10^7^ which benefits from low linear and nonlinear loss in the high-index waveguides. In the calculation, we neglect the differences between pump, signal and idler for the relatively small pump-signal frequency detuning. And the device is designed to be with anomalous dispersion in C-band to get optimal FWM efficiency. The ultra-high overall field enhancement and optimizing dispersion design make cascaded FWM achievable at relative low CW pump power.

### OFC generation with varying FSR spacing

**Figure 4:**
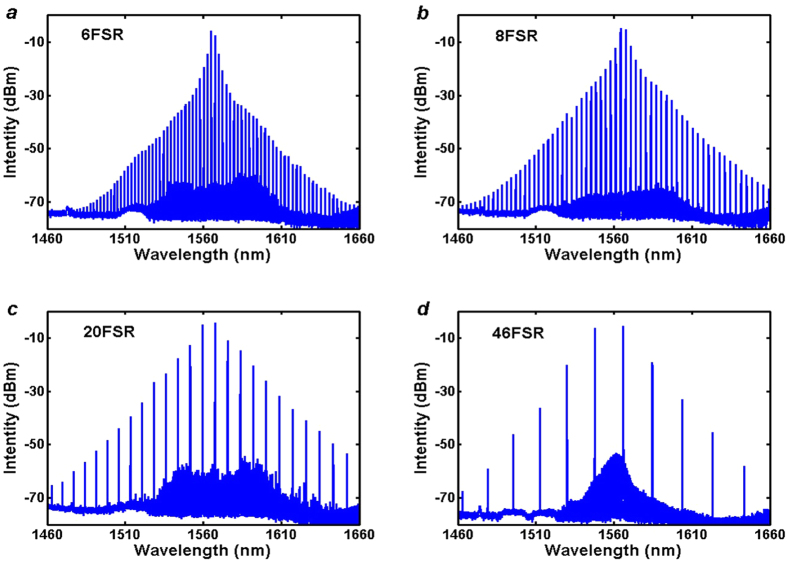
Spectra of generated OFC with different frequency spacing. (**a**) 6-FSR comb, (**b**) 8-FSR comb, (**c**) 20-FSR comb, (**d**) 46-FSR comb.

By fixing the pump wavelength (1567.5 nm) while tuning the signal wavelength, and improving the pump power to around 23dBm (the driven current of the EDFA is ~1050 mA), over 180 nm bandwidth OFCs with frequency spacing varying from 6-FSR to 46-FSR are obtained at the DROP port through a splitter (8:2 beam splitting ratio). Figure 4(a–d) present the OFC spectrum when the signal wavelengths located at 1565.13 nm, 1564.30 nm, 1559.52 nm and 1549.29 nm, corresponding to 6, 8, 20 and 46-fold FSR of the MRR respectively. And the output radiation power of these four OFCs is −2.88 dBm, −1.51 dBm, −1.06 dBm and −2.31 dBm respectively. When the frequency spacing becomes smaller than 6-FSR, similar OFC is also observed. However, its bandwidth gets narrower because of the reduction of FWM efficiency. Meanwhile, we also observed similar OFCs with frequency spacing larger than 3 THz in the experiments. However, the bandwidth of larger spacing OFCs may become narrower because the field enhancement factor reduces due to the waveguide loss increasing caused by the material absorption when wavelength shorter than 1550 nm. So we choose the upper border operating wavelength of the EDFA and programmable filter as the pumps for OFC generation. Another obstacle issue is that extra attenuation should be added in order to balance of the two pumps power because of the non-uniform gain of EDFA and cavity loss; otherwise it would further reduce the bandwidth of the OFC. Even so, it is suggested that larger frequency spacing OFC could be obtained if the EDFA and programmable filter can work in the whole C and L band because the waveguide keeps low loss level up to 1620 nm.

## Discussion

**Figure 5:**
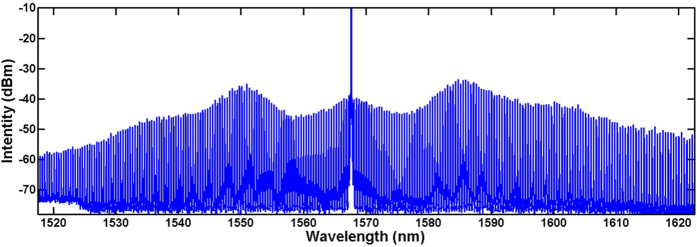
OFC spectrum generated by OPO in the MRR.

The noise-like spectrums around the pump and signal wavelength are generated by optical parametric oscillator (OPO) in the MRR when the pump power exceeds the OPO threshold of the MRR. Its frequency spacing is 49 GHz (FSR of the MRR) and amplitude is much smaller as its lower spectrum gain compared with non-degenerated FWM in the MRR. In order to compare with the single pump OFC, we set the tunable filter with single pass-band centered at 1567.5 nm with 0.4 nm bandwidth. Then single wavelength can be oscillated in the laser loop and used as the pump for OFC generation based on OPO in the MRR. When the pump power reaches the OPO threshold, a comb-like spectrum can be observed. When the pump power reaches 22 dBm, a bandwidth exceeding 150 nm OFC is obtained at the drop port of the MRR, as shown in Fig. 5. We note that the OPO occurs on all the resonances of the MRR even the pump power is just beyond the OPO threshold which is different with situation for the external pump OFC. Another important thing shown in Fig. 4 is the significantly different amplitudes of the generated frequencies as the OFC is directly generated by cascaded FWM in the MRR. This may become an obstacle in some practical applications, such as WDM communication systems which require each channel at the same power level to ensure an equal transmission reaching for all channels. Fortunately, spectral equalization could be realized using multistage parametric mixer which have been verified in wideband flatness OFC generation experiments^27^,^36^.

**Figure 6:**
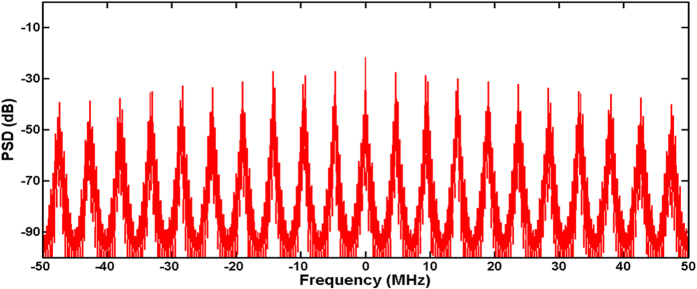
RF beating spectrum measured with a fast photo detector. PSD: Power Spectral Density.

There are multi cavity modes lasing within each MRR resonance in the long cavity for both the dual-pump OFC and single-pump OFC generation experiments. In our OFC generation experiments, there are about 30 main cavity modes lased within each MRR resonance, considering that the total main cavity length is more than 40 meters, yielding a FSR ~4.7 MHz. Figure 6 presents the RF beating spectrum measured with a fast photodetector at the MRR output and shows obvious “super-mode instability”^19^,^37^,^38^. Even undesired, this instability enhances the nonlinear effect in the MRR because the peak power is enhanced by the low-frequency modulation. In order to eliminate the “super-mode instability”, a short cavity can be adopted to realize single main cavity mode oscillation within each MRR resonance to obtain a high optical stability. Compared with high threshold single-pump OFC generation, this scheme is more effective for dual-pump OFC as the low pump power is needed to generate cascaded FWM because of the small cavity gain in such a short laser cavity. Furthermore, our tunable OFC scheme is also an effective method to realize varying repetition rate pulse source which will play an important role in future communication systems.

OFCs with varying frequency spacing can also be realized by externally coupling two coherent pumps into the micro-cavity. Comparing with single pump scheme, it would be much more difficult to couple dual-pump into the micro-cavity simultaneously. This is because the pump fluctuation of two pumps will both induce a thermal drift of the optical resonances and finally cause self-termination^39^. So only several milli-watt pumps power is coupled to the micro-cavity in the previous experiment^31^. In our experiments, the dual-pump is self-oscillated in the laser cavity where the homogeneous gain-broadening of the EDF is suppressed by nonlinear effects (FWM) in MRR at room temperature. This mechanism is the same as the cases of dual- or multi-wavelength EDF Laser realization where mode competition is overcome by nonlinear effect in the laser cavity^40^,^41^. By employing this novel self-locked approach, stable dual-wavelength operation is achieved and their power is equilibrated by adjusting the related attention between the two pumps using the programmable filter. There is no self-termination throughout the experiment for a couple of hours without any thermal or mechanical stabilization methods. So this scheme is very flexible and robust for OFC applications and obviously can also be applied to other type four-port micro-cavities. Moreover, with the development of waveguide amplifiers, on-chip versatile filters and polarization controlling devices, as well as the low-loss coupling technique between the semiconductor gain and waveguides, the proposed system design is potential of realizing the hybrid integration, even monolithically integrated OFC source.

## Conclusion

We propose an OFC generation scheme based on dual-pump FWM in a CMOS-compatible nonlinear micro-ring resonator. The dual pumps are self-oscillated using an amplifying fiber loop combined with an MRR nested in the loop. This design is immune to the thermal or mechanical perturbations due to the intrinsic feedback of the laser loop and self-termination is effectively avoided. These two lasing wavelengths can be tuned flexibly by programming the tunable filter. Meanwhile, benefited from the ultra-high overall field enhancement factor (~9.14 × 10^7^), cascaded FWM effect is observed with low power continuous wave pump and broadband OFC is successfully achieved. Furthermore, the frequency spacing of the OFC can continuously vary from 6 to 46 FSRs which is the highest order spacing multiplication OFC realized in MRR to date to our knowledge. This flexible and robust OFC with varying FSR spacing is a practical chip-based OFC source which has potential applications in future astronomy, microwave photonics, and ultra-high speed optical communication systems.

## Methods

### Device

Figure 1(a) shows the schematic of the four-port high-Q MRR reported here based on a high-index doped silica glass integrated platform which is fabricated by a CMOS compatible process as description in the paper by Razzari, L. *et al*.^42^. The waveguide core is low-loss, high-index (n = 1.6) doped silica glass with 2 × 3 μm waveguide cross-section surrounded by SiO_2_ cladding. The waveguide is optimized with small anomalous dispersion to guarantee a large FWM gain in the C-band. Meanwhile, the waveguides exhibit very low linear propagation loss ~0.06 dB/cm at 1550 nm which is tested by comparing the loss measurement between a short straight waveguide and a 50 cm spiral waveguide on the same chip and negligible nonlinear optical loss^32^. For improving the coupling efficiency between fiber and waveguide, a mode transformer (MT) structure is added to input-output port of our device. Thus our device can be pigtailed directly with fiber array (FA) with a coupling loss between fiber and waveguide lower than 1.0 dB per facet.

## Additional Information

**How to cite this article**: Wang, W. *et al*. Dual-pump Kerr Micro-cavity Optical Frequency Comb with varying FSR spacing. *Sci. Rep*. **6**, 28501; doi: 10.1038/srep28501 (2016).
